# Genetic Variation of Methylenetetrahydrofolate Reductase (*MTHFR*) and Thymidylate Synthase (*TS*) Genes Is Associated with Idiopathic Recurrent Implantation Failure

**DOI:** 10.1371/journal.pone.0160884

**Published:** 2016-08-25

**Authors:** Youngsok Choi, Jung Oh Kim, Sung Han Shim, Yubin Lee, Ji Hyang Kim, Young Joo Jeon, Jung Jae Ko, Woo Sik Lee, Nam Keun Kim

**Affiliations:** 1 Department of Biomedical Science, College of Life Sciences, CHA University, Seongnam, South Korea; 2 Fertility Center of CHA Gangnam Medical Center, CHA University, Seoul, South Korea; 3 Institute for Clinical Research, CHA Bundang Medical Center, School of Medicine, CHA University, Seongnam, South Korea; 4 Department of Obstetrics and Gynecology, CHA Bundang Medical Center, School of Medicine, CHA University, Seongnam, South Korea; Queen's University Belfast, UNITED KINGDOM

## Abstract

The one-carbon metabolism pathway disorder was important role in successful pregnancy. The MTHFR and TS protein were crucial factor in one-carbon metabolism. To investigate the association between recurrent implantation failure (RIF) and enzymes in the one-carbon metabolism pathway. A total of 120 women diagnosed with RIF and 125 control subjects were genotyped for *MTHFR* 677C>T, 1298A>C, *TSER* 2R/3R and *TS* 1494del/ins by a polymerase chain reaction-restriction fragment length polymorphism assay. According to the gene-gene combination analysis, the *MTHFR* 677/*MTHFR* 1298 (TT/AA) and *MTHFR* 677/*TS* 1494 (TT/6bp6bp) genetic combinations were associated with relatively higher risks [adjusted odds ratio (AOR), 2.764; 95% CI, 1.065–7.174; *P* = 0.037 and AOR, 3.186; 95% CI, 1.241–8.178; *P* = 0.016] in RIF patients compared to the CC/AA (*MTHFR* 677/*MTHFR* 1298) and TT/6bp6bp (*MTHFR* 677/*TS* 1494) combinations, respectively. The results suggested that the combined *MTHFR* 677/*MTHFR* 1298 genotype might be associated with increased risk of RIF. To the best of our knowledge, this study is the first to elucidate the potential association of *MTHFR*, *TS* and *TSER* polymorphisms with RIF risk in Korean patients.

## Introduction

Recurrent implantation failure (RIF) is one of the most common reproductive disorders observed at *in vitro* fertilization (IVF) clinics. RIF is defined as implantation failure following three IVF cycles involving the transfer of a high-grade embryo [1]. Various factors influence successful implantation, including anatomic or endometrial factors, thrombophilia, genetics, and immunologic factors, to name a few [2,3]. However, in terms of a clinical approach, the etiology of RIF remains a complicated challenge [2,4].

Successful implantation occurs during a short period of time between days 7 to 10 of the secretory phase of the normal menstrual cycle, when the embryo develops into a blastocyst and migrates to the receptive uterus. Communication between the embryo and the uterus is critical for synchronizing embryonic development and uterine differentiation during the implantation window and is regulated by numerous pathways, including hormones and signaling factors [5]. Among these pathways, folate metabolism is reported to be an essential regulator of early development and pregnancy [6–8]. Folate is a critical molecule in the synthesis of *S*-adenosylmethionine (SAMe), which acts as a methyl group carrier in cellular processes including DNA synthesis, DNA methylation, and amino-acid metabolism. Additionally, folate metabolism is important for folate-homocysteine homeostasis, which is regulated by numerous enzymes in the folate-methionine cycle.

Methylenetetrahydrofolate reductase (MTHFR), which is required for the conversion of 5,10-methylenetetrahydrofolate (5,10-MTHF) to 5-methyltetrahydrofolate (5-MTHF), is one of the major regulatory enzymes in the folate-homocysteine cycle. The methyl group of 5-MTHF is transferred to homocysteine to produce methionine, which is important for the methylation of various substrates such as DNA, RNA, proteins, and lipids. Further, genetic polymorphisms in *MTHFR* are associated with various diseases [9]. Thymidylate synthase (TS), which catalyzes the conversion of deoxyuridine monophosphate (dUMP) to deoxythymidine monophosphate (dTMP) by the transfer of a 5,10-MTHF methyl group, is a crucial enzyme in DNA biosynthesis [10]. As well, the activity of TS is affected by polymorphisms in *TS* and in the *TS* enhancer region (TSER), which likewise results in altered metabolic reactions and the occurrence of disease [11].

In this study, we demonstrated a relationship between RIF and genetic polymorphisms in genes that encode enzymes involved in folate metabolism, including *MTHFR* and *TS*, as well as in the enhancer region of *TS*, in Korean patients experiencing RIF.

## Materials and Methods

### Participants

The study population consisted of 225 female participants recruited from the Department of Obstetrics and Gynecology of CHA Bundang Medical Center, CHA University (Seongnam, Korea) between March 2010 and December 2012 who were experiencing RIF (n = 120) or served as controls (n = 125). The clinical characteristics of the participants are summarized in Table 1. The mean age of the RIF patients and the controls was 34.23 ± 3.33 and 32.75 ± 7.47, respectively (Table 1).

**Table 1:** Clinical characteristics of RIF patients and control subjects.

Characteristic	Control (n = 125)	RIF patients (n = 120)
Age (y, mean± SD)	32.75 ± 7.47	34.23 ± 3.33
BMI (kg/m^2^, mean± SD)	21.72 ± 3.40	20.96 ± 2.59
Previous implantation failure (N, mean± SD)	NA	1.59 ± 0.50
Live birth (N, mean± SD)	1.78 ± 0.74	NA
Mean gestational age (week, mean± SD)	39.36 ± 1.66	NA
Confirmed history of RPL (n)	NA	17 (14.2)
tHcy (μmol/L, mean± SD)	NA	6.56 ± 1.32
Folate (ng/mL, mean± SD)	NA	15.37 ± 11.23
BUN (mg/dl, mean± SD)	NA	10.35 ± 2.87
Creatinine (mg/dl, mean± SD)	NA	0.78 ± 0.1
Uric acid (mg/dl, mean± SD)	NA	3.93 ± 0.96
Total Cholesterol (mg/dl, mean± SD)	NA	188.93 ± 44.28

Note: RIF, recurrent implantation failure; SD, standard deviation; BMI, body mass index; RPL, recurrent pregnancy loss; NA, not applicable; tHcy, total plasma homocysteine; BUN, blood urea nitrogen

The study was approved by the Institutional Review Board of CHA Bundang Medical Center reviewed on 23 February 2010 (reference no. PBC09–120) and all patients provided written informed consent.

Serum human chorionic gonadotropin (hCG) concentrations were less than 5 mIU/mL 14 days after embryo transfer. All transferred embryos were examined by the embryologist before transfer and were deemed to be of good quality. We evaluated both the male and female partners in couples experiencing RIF. Subjects who were diagnosed with RIF due to anatomic, chromosomal, hormonal, infectious, autoimmune, or thrombotic causes were excluded from the study group. Anatomical abnormalities were evaluated using several imaging modalities including sonography, hysterosalpingogram, hysteroscopy, computerized tomography, and magnetic resonance imaging. Karyotyping was conducted using standard protocols. Hormonal causes including hyperprolactinemia, luteal insufficiency, and thyroid disease were excluded by measuring prolactin, thyroid-stimulating hormone, free T4, follicle-stimulating hormone, luteinizing hormone, and progesterone levels in peripheral blood. Lupus anticoagulant and anticardiolipin antibodies were examined to rule out autoimmune diseases such as lupus and antiphospholipid syndrome. Thrombotic disorders were defined as thrombophilia and were evaluated by protein C and protein S deficiencies and by the presence of anti-α2 glycoprotein antibody. Among the initial 167 patients evaluated, 47 who had intrauterine adhesion, hypothyroidism, trisomy and chromosomal translocation (patients or spouses), or antiphospholipid syndrome were excluded from the patient group, leaving 120 patients for the study. Enrollment criteria for the control group included regular menstrual cycles, normal karyotype (46XX), a history of at least one naturally-conceived pregnancy, and no history of pregnancy loss. Data were collected identically for both groups.

### Genotyping

Genomic DNA was extracted from patient peripheral blood samples using a G-DEX(TM) blood extraction kit (iNtRON Biotechnology, Seongnam, South Korea). All of the genetic polymorphisms were detected by polymerase chain reaction (PCR) amplification and restriction enzyme digestion. The PCR primers for each polymorphism were as follows: *MTHFR* 677C>T, forward 5’-TGA AGG AGA AGG TGT CTG CGG GA-3’ and reverse 5’-AGG ACG GTG CGG TGA GAG TC-3’; *MTHFR* 1298A>C, forward 5'-CTT TGG GGA GCT GAA GGA CTACTA C-3' and reverse 5'-CAC TTT GTG ACC ATT CCG GTT TG-3'; *TSER* 2R/3R, forward 5’-CGT GGC TCC TGC GTT TCC-3’ and reverse 5’-GAG CCG GCC ACA GGC ATG-3’; and *TS* 1494 0bp/6bp, forward 5’-CAA ATC TGA GGG AGC TGA GT-3’ and reverse 5’-CAG ATA AGT GGC AGT ACA GA-3’.

The *MTHFR* 677C>T and *MTHFR* 1298A>C polymorphism PCR products were confirmed by restriction enzyme digestion with *Hin*fI and *Fnu*4HI (New England BioLaboratories, Ipswich, MA, USA). For *MTHFR* 677, a 203 bp undigested PCR product indicated the CC genotype, three bands at 203, 173, and 30 bp, respectively, indicated the heterozygous CT genotype, and two bands at 170 and 30 bp, respectively, indicated the homozygous TT genotype. For *MTHFR* 1298, a single band at 138 bp indicated the AA genotype, and two bands at 119 and 19 bp, respectively, indicated the homozygous CC genotype. The *TS* 1494 0bp/6bp polymorphism fragment was 142 bp for the 0bp allele and 148 bp for the 6bp allele. The PCR products were digested with *Dra*I (New England BioLaboratories, Ipswich, MA, USA), resulting in a band at 142 bp (0bp/0bp), three bands at 142 bp, 88 bp, and 60 bp, respectively (0bp/6bp), and two bands at 88 bp and 60 bp, respectively (6bp/6bp). A series of two (2R) or three (3R) 28 bp tandem repeats for the *TSER* 2R/3R polymorphism were confirmed by electrophoretic separation on 4% agarose gels. All of the digestion reactions were performed at 37°C for several hours, depending on the enzymes.

All genotypes were confirmed in triplicate to rule out genotyping errors due to a violation of the Hardy-Weinberg equilibrium (HWE). In addition, some of the PCR products were randomly chosen for DNA sequencing using an ABI 3730xl DNA Analyzer (Applied Biosystems, Foster City, CA, USA).

### Statistical analysis

Differences in genetic frequencies of the polymorphisms between patients and control subjects were compared using Fisher’s exact test and logistic regression. The odds ratio (OR) and 95% confidence interval (CI) were used as a measure of the strength of the association between genotype frequencies and RIF. The OR and 95% CI were also used to assess the relationship between each specific polymorphism and allele combination. The polymorphisms with RIF incidence was calculated using adjusted ORs (AORs) and 95% CIs from logistic regression adjusted for age. Statistical significance was accepted at a level of *P* < 0.05. The false discovery rate (FDR) correction was performed to adjust for multiple comparisons. All of the polymorphisms were in HWE (*P* > 0.05). Statistical analyses were performed using Graphpad Prism 4.0 (Graphpad Software, Inc., San Diego, CA, USA), StatsDirect software version 2.4.4 (StatsDirect Ltd., Altrincham, UK), HaploView 4.1 (Broad Institute of MIT and Harvard, Boston, MA, USA), and HAPSTAT 3.0 (University of North Carolina, Chapel Hill, NC, USA). Gene-gene interaction analysis was performed using the open source multidimensional reduction (MDR) software package v.2.0 (www.epistasis.org). All possible combinations of the polymorphisms were studied using the MDR analysis to determine the combinations with strong synergistic effects.

## Results

The clinical characteristics of all participants are summarized in Table 1. The average number of live births and length of gestation of control subjects were 1.78 ± 0.74 and 39.36 ± 1.66, respectively. RIF patients had no live births and 17 RIF patients had verified histories of recurrent pregnancy loss (Table 1).

To examine the relationship between RIF and polymorphisms in major folate metabolism enzymes and associated genetic enhancer regions, seven alleles were chosen, including *MTHFR* 677, *MTHFR* 1298, *TSER*, and *TS* 1494. The genotype and frequency of the genetic polymorphisms are summarized in Table 2, S1 Table [patients without recurrent pregnancy loss (RPL)] and S2 Table [analysis according to number of previous implantation failure (IF)]. Four of the polymorphisms analyzed were in HWE. The *MTHFR* 677C>T, *MTHFR* 1298A>C, *TSER* 2R/3R, and *TS* 1494 0bp/6bp polymorphisms were not related to the prevalence of RIF.

**Table 2:** Genotype frequencies of one-carbon metabolism-related gene polymorphisms between controls and RIF patients.

Genotype	Controls	RIF patients	Reference allele	Models	AOR (95% CI)	*P*	FDR-*P*
*MTHFR* 677C>T	n = 125	n = 120					
CC	46 (36.8)	35 (29.2)	677C	Additive	1.394 (0.957―2.030)	0.083	0.332
CT	64 (51.2)	60 (50.0)	677C	Dominant	1.384 (0.807―2.375)	0.238	0.476
TT	15 (12.0)	25 (20.8)	677C	Recessive	1.834 (0.908―3.705)	0.091	0.364
HWE *P*	0.308	0.939					
*MTHFR* 1298A>C							
AA	79 (63.2)	78 (65.0)	1298A	Additive	1.005 (0.631―1.600)	0.984	0.984
AC	43 (34.4)	38 (31.7)	1298A	Dominant	0.977 (0.576―1.657)	0.931	0.931
CC	3 (2.4)	4 (3.3)	1298A	Recessive	1.273 (0.277―5.851)	0.756	0.907
HWE *P*	0.306	0.810					
*TSER* 2R/3R							
3R3R	82 (65.6)	81 (67.5)	3R	Additive	0.958 (0.615―1.493)	0.850	0.984
2R3R	37 (29.6)	34 (28.3)	3R	Dominant	0.953 (0.558―1.628)	0.860	0.931
2R2R	6 (4.8)	5 (4.2)	3R	Recessive	0.930 (0.275―3.143)	0.907	0.907
HWE *P*	0.497	0.811					
*TS* 1494 0bp/6bp							
0bp0bp	70 (56.0)	59 (49.2)	14940bp	Additive	1.242 (0.835―1.848)	0.285	0.570
0bp6bp	45 (36.0)	51 (42.5)	14940bp	Dominant	1.391 (0.836―2.316)	0.204	0.476
6bp6bp	10 (8.0)	10 (8.3)	14940bp	Recessive	1.091 (0.435―2.739)	0.852	0.907
HWE *P*	0.471	0.826					

RIF, recurrent implantation failure; AOR, adjusted odds ratio; FDR, false discovery rate. Adjusted by age of female participants.

Combined genotype analysis was performed in *MTHFR* 677/*MTHFR* 1298, *MTHFR* 677/*TSER* 238, *MTHFR* 677/*TS* 1494, *MTHFR* 1298/TSER 238, *MTHFR* 1298/*TS* 1494, and *TSER* 238/*TS* 1494 (Table 3). The combined genotype analysis results that indicated an association with RIF risk included *MTHFR* 677TT/*MTHFR* 1298AA [adjusted odds ratio (AOR), 2.764; 95% CI, 1.0665–7.174; *P* = 0.037], *MTHFR* 677TT/*TS* 1494 0bp6bp+6bp6bp (AOR, 3.185; 95% CI, 1.241–8.178; *P* = 0.016), and *MTHFR* 1298AA/*TS* 1494 0bp6bp+6bp6bp (AOR, 1.945; 95% CI, 1.024–3.694; *P* = 0.042). In addition, the combined genotype analyses, ranked according to the number of implantation failure, are presented in S3 Table. However, the *MTHFR* 1298AA/*TS* 1494 0bp6bp+6bp6bp was not significant in patients without RPL (S4 Table).

**Table 3:** The combination model of one-carbon metabolism-related gene polymorphisms between controls and RIF patients.

1st SNP	2nd SNP	Controls	RIF patients	AOR (95% CI)	*P*	FDR-*P*
*MTHFR* 677C>T	*MTHFR* 1298A>C	n = 125	n = 120			
CC	AA	21 (16.8)	13 (10.8)	1.000 (reference)		
CC	AC	22 (17.6)	18 (15.0)	1.358 (0.530―3.480)	0.523	0.523
CC	CC	3 (2.4)	4 (3.3)	1.994 (0.375―10.612)	0.418	0.523
CT	AA	43 (34.4)	40 (33.3)	1.486 (0.657―3.363)	0.342	0.523
CT	AC	21 (16.8)	20 (16.7)	1.755 (0.662―4.652)	0.258	0.523
TT	AA	15 (12.0)	25 (20.8)	**2.764 (1.065―7.174)**	**0.037**	0.185
*MTHFR* 677C>T	*TSER* 2R/3R					
CC+CT	3R3R	70 (56.0)	65 (54.2)	1.000 (reference)		
CC+CT	2R3R+2R2R	40 (32.0)	30 (25.0)	0.843 (0.468―1.520)	0.570	0.570
TT	3R3R	12 (9.6)	16 (13.3)	1.388 (0.607―3.172)	0.437	0.570
TT	2R3R+2R2R	3 (2.4)	9 (7.5)	3.195 (0.823―12.407)	0.093	0.279
*MTHFR* 677C>T	*TS* 1494 0bp/6bp					
CC+CT	0bp0bp	62 (49.6)	53 (44.2)	1.000 (reference)		
CC+CT	0bp6bp+6bp6bp	48 (38.4)	42 (35.0)	1.094 (0.623―1.920)	0.754	0.858
TT	0bp0bp	8 (6.4)	6 (5.0)	0.902 (0.291―2.793)	0.858	0.858
TT	0bp6bp+6bp6bp	7 (5.6)	19 (15.8)	**3.186 (1.241―8.178)**	**0.016**	0.048
*MTHFR* 1298A>C	*TSER* 2R/3R					
AA	3R3R	53 (42.4)	51 (42.5)	1.000 (reference)		
AA	2R3R+2R2R	26 (20.8)	27 (22.5)	1.087 (0.559―2.114)	0.805	0.805
AC+CC	3R3R	29 (23.2)	30 (25.0)	1.123 (0.589―2.142)	0.725	0.805
AC+CC	2R3R+2R2R	17 (13.6)	12 (10.0)	0.738 (0.319―1.708)	0.478	0.805
*MTHFR* 1298A>C	*TS* 1494 0bp/6bp					
AA	0bp0bp	47 (37.6)	34 (28.3)	1.000 (reference)		
AA	0bp6bp+6bp6bp	32 (25.6)	44 (36.7)	**1.945 (1.024―3.694)**	**0.042**	0.126
AC+CC	0bp0bp	23 (18.4)	25 (20.8)	1.484 (0.718―3.066)	0.287	0.431
AC+CC	0bp6bp+6bp6bp	23 (18.4)	17 (14.2)	1.012 (0.466―2.198)	0.975	0.975
*TSER* 2R/3R	*TS* 1494 0bp/6bp					
3R3R	0bp0bp	53 (42.4)	45 (37.5)	1.000 (reference)		
3R3R	0bp6bp+6bp6bp	29 (23.2)	36 (30.0)	1.530 (0.809―2.892)	0.191	0.573
2R3R+2R2R	0bp0bp	17 (13.6)	14 (11.7)	0.955 (0.423―2.158)	0.912	0.912
2R3R+2R2R	0bp6bp+6bp6bp	26 (20.8)	25 (20.8)	1.166 (0.589―2.309)	0.660	0.912

Adjusted by age of female participants.

RIF, recurrent implantation failure; SNP, single nucleotide polymorphism; AOR, adjusted odds ratio

To investigate the allele combinations of the *MTHFR* 677, *MTHFR* 1298, *TS* 1494, and *TSER* polymorphisms, we carried out gene-gene interaction analysis using the haplotype-based MDR method (Table 4). The results of the MDR analysis revealed that allele combinations increased the relative risk of RIF. The *MTHFR* 677/*MTHFR* 1298/*TSER*/*TS* 1494 allele combination (C-A-3R-6bp and T-A-2R-6bp) was significantly higher in RIF patients than in control subjects. The OR of the combined polymorphisms (C-A-3R-6bp) was 3.678 (95% CI, 1.363–9.929) and the OR of T-A-2R-6bp was 2.885 (95% CI, 1.222–6.814) when the reference combination was C-A-3R-0bp. In addition, the *MTHFR* 677/*MTHFR* 1298/*TSER* 238, *MTHFR* 677/*MTHFR* 1298/*TS* 1494, *MTHFR* 677/*TSER* 238/*TS* 1494, and *MTHFR* 1298/*TSER* 238/*TS* 1494 allele combinations increased the risk of RIF. The OR of the combined alleles (T-A-2R) was 2.407 (95% CI, 1.166–4.970) in the *MTHFR* 677/*MTHFR* 1298/*TSER* 238 combination. As well, the T-A-0bp (*MTHFR* 677/*MTHFR* 1298/*TS* 1494) increased the risk of RIF (OR, 1.964; 95% CI, 1.109–3.478). Further, the T-2R-6bp (*MTHFR* 677/*TSER* 238/*TS* 1494) and the A-3R-6bp (*MTHFR* 1298/*TSER* 238/*TS* 1494) allele combinations had higher ORs of 2.395 (95% CI, 1.036–5.534) and 1.788 (95% CI, 1.029–3.107), respectively. In particular, the risk of RIF in patients carrying the combined *MTHFR* 677/*MTHFR* 1298/*TSER* 238/*TS* 1494 (C-A-3R-6bp) or *MTHFR* 677/*MTHFR* 1298/*TSER* 238 (T-A-2R) alleles increased 3.7 and 2.4 times, respectively, compared to women with the C-A-3R-0bp or C-A-3R reference alleles. The risk of RIF was not altered when the reference was the value combined, except in the self in haplotype-based MDR analysis. In contrast, the *MTHFR* 677/*MTHFR* 1298/*TSER* 238/*TS* 1494 (C-A-2R-6bp) and *MTHFR* 677/*MTHFR* 1298/*TS* 1494 (C-A-2R) allele combinations exhibited protective effects compared to the reference allele combinations. The ORs of C-A-2R-6bp and C-2R-6bp were 0.045 (95% CI, 0.003–0.765) and 0.345 (95% CI, 0.147–0.812), respectively (Table 4), which suggested that patients with C-A-2R-6bp allele combination reduced risk of RIF. In addition, the *MTHFR* 677/*TSER* 238 (C-2R and T-2R) was significantly association with RIF risk (S5 Table).

**Table 4:** Allelic gene-gene interaction of one-carbon metabolism-related gene polymorphisms between controls and RIF patients.

Haplotype	Controls (2n = 250)	RIF patients (2n = 240)	OR (95% CI)	*P*	FDR-*P*
*MTHFR* 677/*MTHFR* 1298/*TSER* 238/*TS* 1494					
C-A-3R-0bp	74 (29.7)	57 (23.8)	1.000 (reference)		
C-A-3R-6bp	6 (2.5)	17 (7.1)	**3.678 (1.363–9.929)**	**0.012**	0.055
C-A-2R-0bp	13 (5.2)	10 (4.2)	0.999 (0.408–2.442)	1.000	1.000
C-A-2R-6bp	14 (5.4)	0 (0.0)	**0.045 (0.003–0.765)**	**0.001**	0.011
C-C-3R-0bp	24 (9.7)	32 (13.3)	1.731 (0.920–3.257)	0.110	0.242
C-C-3R-6bp	13 (5.0)	6 (2.7)	0.599 (0.215–1.674)	0.457	0.628
C-C-2R-0bp	9 (3.5)	4 (1.6)	0.577 (0.169–1.970)	0.558	0.682
C-C-2R-6bp	4 (1.4)	4 (1.7)	1.298 (0.311–5.418)	0.730	0.803
T-A-3R-0bp	64 (25.6)	61 (25.2)	1.237 (0.756–2.025)	0.452	0.628
T-A-3R-6bp	20 (7.9)	23 (9.7)	1.493 (0.748–2.982)	0.292	0.535
T-A-2R-0bp	1 (0.4)	6 (2.4)	7.789 (0.911–66.57)	0.047	0.129
T-A-2R-6bp	9 (3.7)	20 (8.5)	**2.885 (1.222–6.814)**	**0.015**	0.055
*MTHFR* 677/*MTHFR* 1298/*TSER* 238					
C-A-3R	82 (32.8)	76 (31.8)	1.000 (reference)		
C-A-2R	25 (10.0)	8 (3.2)	**0.345 (0.147–0.812)**	**0.013**	0.058
C-C-3R	38 (15.3)	39 (16.1)	1.107 (0.642–1.910)	0.781	0.781
C-C-2R	11 (4.3)	7 (3.1)	0.687 (0.253–1.863)	0.619	0.781
T-A-3R	81 (32.3)	81 (33.8)	1.079 (0.696–1.673)	0.739	0.781
T-A-2R	13 (5.3)	29 (12.1)	**2.407 (1.166–4.970)**	**0.023**	0.058
*MTHFR* 677/*MTHFR* 1298/*TS* 1494					
C-A-0bp	85 (34.0)	68 (28.3)	1.000 (reference)		
C-A-6bp	22 (8.8)	16 (6.7)	0.909 (0.443–1.865)	0.856	0.856
C-C-0bp	35 (13.8)	36 (14.8)	1.286 (0.731–2.260)	0.392	0.675
C-C-6bp	14 (5.8)	10 (4.4)	0.893 (0.373–2.136)	0.829	0.856
T-A-0bp	66 (26.2)	65 (27.3)	1.231 (0.771–1.966)	0.405	0.675
T-A-6bp	28 (11.4)	44 (18.5)	**1.964 (1.109–3.478)**	**0.023**	0.115
*MTHFR* 677/*TSER* 238/*TS* 1494					
C-3R-0bp	97 (39.0)	90 (37.5)	1.000 (reference)		
C-3R-6bp	19 (7.6)	22 (9.0)	1.248 (0.634–2.458)	0.606	0.707
C-2R-0bp	23 (9.1)	13 (5.4)	0.609 (0.291–1.275)	0.205	0.359
C-2R-6bp	17 (6.8)	6 (2.3)	0.380 (0.144–1.008)	0.049	0.114
T-3R-0bp	65 (26.0)	60 (25.1)	0.995 (0.632–1.565)	1.000	1.000
T-3R-6bp	20 (7.8)	24 (10.1)	1.293 (0.669–2.501)	0.504	0.706
T-2R-0bp	0 (0.0)	6 (2.5)	14.01 (0.777–252.3)	0.014	0.098
T-2R-6bp	9 (3.8)	20 (8.2)	**2.395 (1.036–5.534)**	**0.046**	0.114
*MTHFR* 1298/*TSER* 238/*TS* 1494					
A-3R-0bp	136 (54.4)	117 (48.7)	1.000 (reference)		
A-3R-6bp	26 (10.5)	40 (16.5)	**1.788 (1.029–3.107)**	**0.039**	0.273
A-2R-0bp	16 (6.3)	16 (6.7)	1.162 (0.557–2.426)	0.711	0.749
A-2R-6bp	23 (9.2)	22 (9.0)	1.112 (0.589–2.098)	0.749	0.749
C-3R-0bp	26 (10.5)	34 (14.0)	1.520 (0.862–2.681)	0.154	0.539
C-3R-6bp	12 (5.0)	6 (2.5)	0.581 (0.212–1.597)	0.335	0.620
C-2R-0bp	7 (2.8)	3 (1.1)	0.498 (0.126–1.971)	0.354	0.620
C-2R-6bp	3 (1.3)	4 (1.6)	1.550 (0.340–7.069)	0.708	0.749

RIF, recurrent implantation failure; OR, odds ratio; *p*-value Fisher’s exact test.

**Fig 1:**
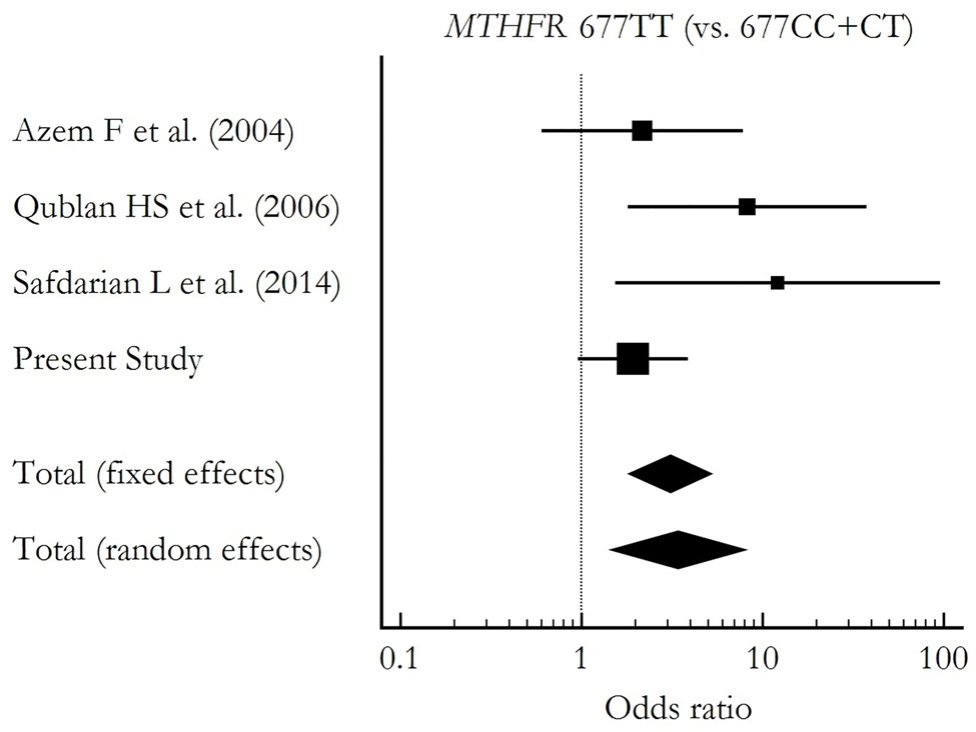
A meta-analysis of *MTHFR* 677C>T in RIF. A meta-analysis of the association between carriers of the T allele (individuals with TT genotype) in the *MTHFR* 677C>T polymorphism and recurrent implantation failure (RIF). The fixed and random effects models were used to calculate the pooled weighted odds ratios (ORs).

The results of the *MTHFR* 677C>T screening is summarized in Fig 1. We were search association study between *MTHFR* 677C>T polymorphism and RIF, found 3 studies in PubMed database (**http://www.ncbi.nlm.nih.gov/pubmed**). A total of 364 controls and 351 patients were positive for the *MTHFR* 677C>T polymorphism. Overall, the results indicate an association of this polymorphism with risk of RIF (OR, 3.394; 95% CI, 1.451–7.938; Fig 1, S6 Table).

## Discussion

At present, the relationship between RIF and polymorphisms in genes that encode major folate metabolism enzymes remains unclear. However, in this study, we demonstrated that patients carrying the *MTHFR* 677 and *MTHFR* 1298 alleles in combination with the *TS* 1494 polymorphism had a significantly higher risk of experiencing RIF.

Successful implantation begins with proper implantation of the embryo in a receptive uterine endometrium. Afterward, the endometrium undergoes dynamic morphological and functional changes to become receptive to the embryo during early implantation [12]. In addition, the uterus undergoes cellular processes, including DNA synthesis and angiogenesis, which are required for cell proliferation and decidualization, and for orchestrating complicated implantation processes. Hence, DNA synthesis and homeostasis are important during this process. As well, folate acts as a methyl group carrier for the targets and is required for the homeostasis of the one-carbon metabolism pathway. One of the forms of folate is 5,10-MTHF, which can be used for the synthesis of either methionine or dTMP. Methionine is synthesized by the transfer of a 5-MTHF methyl group to homocysteine, whereas dTMP is generated by the transfer of a 5-MTHF methyl group to deoxyuridine monophosphate (dUTP) [13].

The one-carbon metabolism pathway is precisely regulated by two critical enzymes, MTHFR and TS. Further, MTHFR generates 5-MTHF from 5,10-MTHF [13,14], and is located on the short arm of chromosome 1 (1p36.3[15]. *MTHFR* 677C>T, a polymorphism of *MTHFR*, leads to the conversion of the amino acid alanine to valine, resulting in decreased *MTHFR* activity and impaired enzyme activity [16]. Also, the *MTHFR* 677C>T polymorphism is associated with various diseases, including stroke, hypertension, and cancer [reviewed in [9,17]]. The A and C nucleotides are involved in the *MTHFR* 1298 polymorphism, but the *MTHFR* 1298A allele is more common than the 1298C allele. The *MTHFR* 1298A>C polymorphism leads to the substitution of alanine for glutamine at amino acid 429, but the mutation does not affect the thermolability or FAD release activity functions of the MTHFR protein, or the protective effects of 5-methyl-THF [18]. Several recent reports have demonstrated an association between the one-carbon metabolism enzymes and recurrent spontaneous abortion [19–21]. In addition, idiopathic infertile women exhibit an increased frequency of *MTHFR* 677C>T polymorphisms compared to control women [22]. Safdarian et al. reported association between *MTHFR* 677C>T and RIF risk in term of hereditary thrombophilia [23].

Thymidylate synthetase catalyzes the conversion of dUMP to dTMP by oxidation of 5,10-MTHF [24]. As well, dTMP is required for *de novo* DNA synthesis. Further, two *TSER* and *TS* 1494 polymorphisms affect the transcription and translation of the *TS* gene [25]. The 5’-untranslated region (UTR) of TSER contains 2R and 3R repeats of 28 bp sequences, and the 3’-UTR of *TS* 1494 has either a 6 bp deletion or insertion, which results in the modulation of *TS* expression and stability [25–28]. However, little is known regarding the effect of *TSER* and *TS* polymorphisms on RIF. Recently, we reported that the TSER 2R2R and *TS* 6bp6bp combined genotype was associated with cancer [29], which suggests that the presence of these combinations might affect susceptibility to RIF.

The results of our previous studies established an association between polymorphisms in folate metabolism-related genes (*MTHFR* 677C>T, 1298A>C, *TSER* 2R/3R, and *TS* 1494 0bp/6bpins/del) and increased risk of reproductive diseases, including RPL, premature ovarian failure, and spontaneously aborted embryos in the Korean population [30–34]. However, these studies only showed the difference in genotype frequencies between control subjects and patient groups. In addition, we have identified an indirect effect of the *MTHFR* 677C>T, 1298A>C, and *TSER* 2R/3R polymorphisms on RPL [35]. In previous study, *MTHFR* gene polymorphisms (677C>T and 1298A>C) were reported association between maternal, fetal and paternal in RPL risk by meta-analysis [36]. These meta-analyses of RPL and our screening data of RIF were suggestion that MTHFR 677C>T was considered to crucial genetic factor during implantation, or maintaining pregnancy. In this study, one interesting result was the indication that the *MTHFR* 677TT/1298AA and *MTHFR* 677TT/*TS* 1494 0bp6bp+6bp6bp combinations conveyed increased risk of RIF occurrence. In addition, we identified relationships between the *MTHFR/TSER/TS* genetic polymorphisms and risk of RIF in Korean women. The genetic combinations of *MTHFR* 677/*MTHFR* 1298/*TSER*, TS 1494 (C-A-3R-6bp), and *MTHFR* 677/*MTHFR* 1298/*TS* 1494(C-A-6bp) increased the risk of RIF compared to the risk associated with each reference combination.

We also performed a screening of published studies to investigate the genetic association between *MTHFR* 677C>T and the risk of RIF, which revealed that *MTHFR* 677C>T increased the risk of RIF. However, this study has some limitations. First, the lack of clinical parameters, such as vitamin B6, inflammatory cytokine and hormone levels in RIF women remains to be investigated. Second, the sample size (number of included studies) was so small; therefore, we cannot rule out the possibility of the results being biased, although no significant publication bias was found.

In conclusion, these interesting findings indicated that the combined *MTHFR*, *TSER*, and *TS* genotypes could potentially be novel diagnostic markers for evaluating the risk of experiencing RIF. However, due to the small number of patients and clinical insufficiency, further studies are required to confirm our conclusions. Nonetheless, the results of the current study provided us with a better understanding of idiopathic RIF and the relationship between RIF and polymorphisms in genes that encode major folate metabolism enzymes.

## Supporting Information

S1 Table Genotype frequencies of one-carbon metabolism-related gene polymorphisms between controls and RIF patients without RPL. (DOCX)

S2 Table Genotype frequencies of one-carbon metabolism-related gene polymorphisms between controls and RIF patients according to IF numbers. (DOCX)

S3 Table The combination model of one-carbon metabolism-related gene polymorphisms between controls and RIF patients according to IF numbers. (DOCX)

S4 Table The combination model of one-carbon metabolism-related gene polymorphisms between controls and RIF patients without RPL. (DOCX)

S5 Table Allelic gene-gene interaction (2 sites) of one-carbon metabolism-related gene polymorphisms between controls and RIF patients. (DOCX)

S6 Table Previous and present study for screening of *MTHFR* 677C>T polymorphisms in RIF. (DOCX)
