# Supplementary material for: Genetic Variation of Methylenetetrahydrofolate Reductase (MTHFR) and Thymidylate Synthase (TS) Genes Is Associated with Idiopathic Recurrent Implantation Failure
Source: PLoS One. 2016 Aug 25;11(8):e0160884. doi: 10.1371/journal.pone.0160884 (PMC4999086; doi:10.1371/journal.pone.0160884)
Supplement: S1 Table — (DOCX) [file pone.0160884.s001.docx]

| S1 Table. Genotype frequencies of one-carbon metabolism-related gene polymorphisms between controls and RIF patients without RPL. | | | | | | | | |
| --- | --- | --- | --- | --- | --- | --- | --- | --- |
| Genotype | Controls | RIF patients | Reference allele | Models | AOR (95% CI) | *P* | FDR-*P* | Statistical power |
| *MTHFR* 677C>T | n = 125 | n = 103 |  |  |  |  |  |  |
| CC | 46 (36.8) | 31 (30.1) | 677C | Additive | 1.375 (0.932―2.028) | 0.108 | 0.432 | 25.4% |
| CT | 64 (51.2) | 50 (48.5) | 677C | Dominant | 1.328 (0.759―2.324) | 0.321 | 0.642 | 8.7% |
| TT | 15 (12.0) | 22 (21.4) | 677C | Recessive | 1.878 (0.911―3.873) | 0.088 | 0.352 | 57.2% |
| HWE *P* | 0.308 | 0.826 |  |  |  |  |  |  |
| *MTHFR* 1298A>C |  |  |  |  |  |  |  |  |
| AA | 79 (63.2) | 66 (64.1) | 1298A | Additive | 1.029 (0.632―1.676) | 0.908 | 0.908 | 5.2% |
| AC | 43 (34.4) | 34 (33.0) | 1298A | Dominant | 1.025 (0.592―1.775) | 0.931 | 0.931 | 6.2% |
| CC | 3 (2.4) | 3 (2.9) | 1298A | Recessive | 1.111 (0.218―5.666) | 0.899 | 0.899 | 2.8% |
| HWE *P* | 0.306 | 0.578 |  |  |  |  |  |  |
| *TSER* 2R/3R |  |  |  |  |  |  |  |  |
| 3R3R | 82 (65.6) | 70 (68.0) | 3R | Additive | 0.905 (0.564―1.453) | 0.680 | 0.907 | 8.5% |
| 2R3R | 37 (29.6) | 30 (29.1) | 3R | Dominant | 0.933 (0.534―1.632) | 0.809 | 0.931 | 4.5% |
| 2R2R | 6 (4.8) | 3 (2.9) | 3R | Recessive | 0.650 (0.158―2.679) | 0.551 | 0.899 | 11.1% |
| HWE *P* | 0.497 | 0.921 |  |  |  |  |  |  |
| *TS* 1494 0bp/6bp |  |  |  |  |  |  |  |  |
| 0bp0bp | 70 (56.0) | 51 (49.5) | 14940bp | Additive | 1.241 (0.823―1.871) | 0.303 | 0.606 | 22.5% |
| 0bp6bp | 45 (36.0) | 43 (41.7) | 14940bp | Dominant | 1.373 (0.808―2.333) | 0.242 | 0.642 | 19.8% |
| 6bp6bp | 10 (8.0) | 9 (8.7) | 14940bp | Recessive | 1.149 (0.446―2.960) | 0.773 | 0.899 | 4.7% |
| HWE *P* | 0.471 | 0.988 |  |  |  |  |  |  |
| Adjusted by age of female participants; RIF, recurrent implantation failure; RPL, recurrent pregnancy loss. | | | | | | | | |
