# Supplementary material for: Genetic Variation of Methylenetetrahydrofolate Reductase (MTHFR) and Thymidylate Synthase (TS) Genes Is Associated with Idiopathic Recurrent Implantation Failure
Source: PLoS One. 2016 Aug 25;11(8):e0160884. doi: 10.1371/journal.pone.0160884 (PMC4999086; doi:10.1371/journal.pone.0160884)
Supplement: S2 Table — (DOCX) [file pone.0160884.s002.docx]

| S2 Table. Genotype frequencies of one-carbon metabolism-related gene polymorphisms between controls and RIF patients according to IF numbers. | | | | | | | | | | | |
| --- | --- | --- | --- | --- | --- | --- | --- | --- | --- | --- | --- |
| Genotype | Controls | IF≥3 | Reference allele | Models | AOR (95% CI) | *P* | IF≥4 | Reference allele | Models | AOR (95% CI) | *P* |
| *MTHFR* 677C>T | n=125 | n=107 |  |  |  |  | n=75 |  |  |  |  |
| CC | 46 (36.8) | 33 (30.8) | 677C | Additive | 1.323 (0.900―1.946) | 0.155 | 21 (28.0) | 677C | Additive | 1.489 (0.969―2.286) | 0.069 |
| CT | 64 (51.2) | 52 (48.6) | 677C | Dominant | 1.271 (0.731―2.210) | 0.395 | 37 (49.3) | 677C | Dominant | 1.470 (0.786―2.748) | 0.228 |
| TT | 15 (12.0) | 22 (20.6) | 677C | Recessive | 1.774 (0.860―3.658) | 0.121 | 17 (22.7) | 677C | Recessive | 2.047 (0.9474.427) | 0.069 |
| HWE *P* | 0.308 |  |  |  |  |  |  |  |  |  |  |
| *MTHFR* 1298A>C |  |  |  |  |  |  |  |  |  |  |  |
| AA | 79 (63.2) | 69 (64.5) | 1298A | Additive | 1.010 (0.622―1.639) | 0.968 | 51 (68.0) | 1298A | Additive | 0.941 (0.552―1.605) | 0.069 |
| AC | 43 (34.4) | 35 (32.7) | 1298A | Dominant | 1.009 (0.585―1.738) | 0.976 | 21 (28.0) | 1298A | Dominant | 0.870 (0.470―1.609) | 0.656 |
| CC | 3 (2.4) | 3 (2.8) | 1298A | Recessive | 1.036 (0.203―5.290) | 0.966 | 3 (4.0) | 1298A | Recessive | 1.529 (0.298―7.832) | 0.611 |
| HWE *P* | 0.306 |  |  |  |  |  |  |  |  |  |  |
| *TSER* 2R/3R |  |  |  |  |  |  |  |  |  |  |  |
| 3R3R | 82 (65.6) | 72 (67.3) | 3R | Additive | 0.989 (0.628―1.557) | 0.963 |  | 3R | Additive | 1.111 (0.6379―1.820) | 0.675 |
| 2R3R | 37 (29.6) | 30 (28.0) | 3R | Dominant | 0.972 (0.559―1.690) | 0.920 | 23 (30.7) | 3R | Dominant | 1.130 (0.617―2.069) | 0.693 |
| 2R2R | 6 (4.8) | 5 (4.7) | 3R | Recessive | 1.060 (0.313―3.593) | 0.925 | 4 (5.3) | 3R | Recessive | 1.187 (0.323―4.364) | 0.767 |
| HWE *P* | 0.497 |  |  |  |  |  | 0 (0.0) |  |  |  |  |
| *TS* 1494ins6/del6 |  |  |  |  |  |  |  |  |  |  |  |
| del6del6 | 70 (56.0) | 52 (48.6) | 1494del6 | Additive | 1.242 (0.822―1.877) | 0.303 | 34 (45.3) | 1494del6 | Additive | 1.377 (0.874―2.170) | 0.167 |
| ins6del6 | 45 (36.0) | 47 (43.9) | 1494del6 | Dominant | 1.440 (0.851―2.438) | 0.175 | 35 (46.7) | 1494del6 | Dominant | 1.688 (0.936―3.047) | 0.082 |
| ins6ins6 | 10 (8.0) | 8 (7.5) | 1494del6 | Recessive | 0.957 (0.362―2.535) | 0.930 | 6 (8.0) | 1494del6 | Recessive | 1.029 (0.356―2.970) | 0.959 |
| HWE *P* | 0.471 |  |  |  |  |  |  |  |  |  |  |
| *P*-value by Fisher’s exact test; RIF, recurrent implantation failure; IF, implantation failure | | | | | | |  |  |  |  |  |
