# Supplementary material for: Genetic Variation of Methylenetetrahydrofolate Reductase (MTHFR) and Thymidylate Synthase (TS) Genes Is Associated with Idiopathic Recurrent Implantation Failure
Source: PLoS One. 2016 Aug 25;11(8):e0160884. doi: 10.1371/journal.pone.0160884 (PMC4999086; doi:10.1371/journal.pone.0160884)
Supplement: S3 Table — (DOCX) [file pone.0160884.s003.docx]

| S3 Table. The combination model of one-carbon metabolism-related gene polymorphisms between controls and RIF patients according to IF numbers.. | | | | | | | | |
| --- | --- | --- | --- | --- | --- | --- | --- | --- |
| 1st SNP | 2nd SNP | Controls | IF≥3 | AOR (95% CI) | *P* | IF≥4 | AOR (95% CI) | *P* |
| *MTHFR* 677C>T | *MTHFR* 1298A>C | n=125 | n=107 |  |  | n=75 |  |  |
| CC | AA | 21 (16.8) | 13 (12.1) | 1.000 (reference) |  | 7 (9.3) | 1.000 (reference) |  |
| CC | AC | 22 (17.6) | 3 (2.8) | 1.281 (0.497-3.305) | 0.608 | 3 (4.0) | 1.584 (0.508-4.944) | 0.428 |
| CC | CC | 3 (2.4) | 34 (31.8) | 1.456 (0.247-8.590) | 0.678 | 27 (36.0) | 2.665 (0.424-16.758) | 0.296 |
| CT | AA | 43 (34.4) | 18 (16.8) | 1.260 (0.551-2.883) | 0.584 | 10 (13.3) | 1.852 (0.692-4.957) | 0.220 |
| CT | AC | 21 (16.8) | 22 (20.6) | 1.569 (0.586-4.206) | 0.370 | 17 (22.7) | 1.867 (0.549-6.350) | 0.317 |
| TT | AA | 15 (12.0) | 27 (25.2) | 2.409 (0.917-6.332) | 0.075 | 21 (28.0) | **3.486 (1.146-10.606)** | **0.028** |
| *MTHFR* 677C>T | *TSER* 2R/3R |  |  |  |  |  |  |  |
| CC+CT | 3R3R | 70 (56.0) | 58 (54.2) |  |  | 37 (49.3) |  |  |
| CC+CT | 2R3R+2R2R | 40 (32.0) | 14 (13.1) | 0.860 (0.468-1.581) | 0.628 | 11 (14.7) | 1.051 (0.537-2.056) | 0.886 |
| TT | 3R3R | 12 (9.6) | 8 (7.5) | 1.341 (0.571-3.151) | 0.501 | 6 (8.0) | 1.663 (0.664-4.162) | 0.278 |
| TT | 2R3R+2R2R | 3 (2.4) | 38 (35.5) | 3.152 (0.792-12.542) | 0.103 | 29 (38.7) | 3.916 (0.920-16.667) | 0.065 |
| *MTHFR* 677C>T | *TS* 1494ins6/del6 |  |  |  |  |  |  |  |
| CC+CT | del6del6 | 62 (49.6) | 47 (43.9) |  |  | 29 (38.7) |  |  |
| CC+CT | ins6del6+ins6ins6 | 48 (38.4) | 5 (4.7) | 1.124 (0.629-2.009) | 0.693 | 5 (6.7) | 1.436 (0.746-2.763) | 0.279 |
| TT | del6del6 | 8 (6.4) | 17 (15.9) | 0.839 (0.254-2.768) | 0.773 | 12 (16.0) | 1.356 (0.401-4.580) | 0.624 |
| TT | ins6del6+ins6ins6 | 7 (5.6) | 23 (21.5) | **3.212 (1.229-8.395)** | **0.017** | 18 (24.0) | **3.791 (1.344-10.691)** | **0.012** |
| *MTHFR* 1298A>C | *TSER* 2R/3R |  |  |  |  |  |  |  |
| AA | 3R3R | 53 (42.4) | 46 (43.0) |  |  | 33 (44.0) |  |  |
| AA | 2R3R+2R2R | 26 (20.8) | 26 (24.3) | 1.034 (0.518-2.062) | 0.925 | 15 (20.0) | 1.133 (0.536-2.393) | 0.744 |
| AC+CC | 3R3R | 29 (23.2) | 12 (11.2) | 1.092 (0.560-2.132) | 0.796 | 9 (12.0) | 0.885 (0.409-1.916) | 0.756 |
| AC+CC | 2R3R+2R2R | 17 (13.6) | 39 (36.4) | 0.827 (0.355-1.925) | 0.659 | 31 (41.3) | 0.869 (0.344-2.197) | 0.767 |
| *MTHFR* 1298A>C | *TS* 1494ins6/del6 |  |  |  |  |  |  |  |
| AA | del6del6 | 47 (37.6) | 30 (28.0) |  |  | 20 (26.7) |  |  |
| AA | ins6del6+ins6ins6 | 32 (25.6) | 22 (20.6) | **1.975 (1.017-3.835)** | **0.045** | 14 (18.7) | **2.429 (1.158-5.092)** | **0.019** |
| AC+CC | del6del6 | 23 (18.4) | 16 (15.0) | 1.493 (0.704-3.164) | 0.296 | 10 (13.3) | 1.442 (0.608-3.420) | 0.406 |
| AC+CC | ins6del6+ins6ins6 | 23 (18.4) | 33 (30.8) | 1.089 (0.491-2.412) | 0.834 | 23 (30.7) | 1.035 (0.409-2.616) | 0.942 |
| *TSER* 2R/3R | *TS* 1494ins6/del6 |  |  |  |  |  |  |  |
| 3R3R | del6del6 | 53 (42.4) | 39 (36.4) |  |  | 25 (33.3) |  |  |
| 3R3R | ins6del6+ins6ins6 | 29 (23.2) | 13 (12.1) | 1.653 (0.856-3.193) | 0.135 | 9 (12.0) | 1.848 (0.878-3.892) | 0.106 |
| 2R3R+2R2R | del6del6 | 17 (13.6) | 22 (20.6) | 1.028 (0.444-2.376) | 0.949 | 18 (24.0) | 1.120 (0.435-2.882) | 0.815 |
| 2R3R+2R2R | ins6del6+ins6ins6 | 26 (20.8) | 0 (0.0) | 1.207 (0.594-2.456) | 0.603 | 0 (0.0) | 1.588 (0.726-3.469) | 0.247 |
| Adjusted by age of female participants; RIF, recurrent implantation failure; IF, implantation failure.. | | | | | |  |  |  |
