# Supplementary material for: Genetic Variation of Methylenetetrahydrofolate Reductase (MTHFR) and Thymidylate Synthase (TS) Genes Is Associated with Idiopathic Recurrent Implantation Failure
Source: PLoS One. 2016 Aug 25;11(8):e0160884. doi: 10.1371/journal.pone.0160884 (PMC4999086; doi:10.1371/journal.pone.0160884)
Supplement: S4 Table — (DOCX) [file pone.0160884.s004.docx]

| S4 Table. The combination model of one-carbon metabolism-related gene polymorphisms between controls and RIF patients without RPL. | | | | | | |
| --- | --- | --- | --- | --- | --- | --- |
| 1st SNP | 2nd SNP | Controls | RIF patients | AOR (95% CI) | *P* | FDR-*P* |
| *MTHFR* 677C>T | *MTHFR* 1298A>C | n = 125 | n = 103 |  |  |  |
| CC | AA | 21 (16.8) | 11 (10.7) | 1.000 (reference) |  |  |
| CC | AC | 22 (17.6) | 17 (16.5) | 1.539 (0.579―4.097) | 0.388 | 0.493 |
| CC | CC | 3 (2.4) | 3 (2.9) | 1.734 (0.292―10.30) | 0.545 | 0.545 |
| CT | AA | 43 (34.4) | 33 (32.0) | 1.454 (0.615―3.438) | 0.394 | 0.493 |
| CT | AC | 21 (16.8) | 17 (16.5) | 1.879 (0.671―5.258) | 0.230 | 0.493 |
| TT | AA | 15 (12.0) | 22 (21.4) | 2.838 (1.053―7.653) | 0.039 | 0.195 |
| *MTHFR* 677C>T | *TSER* 2R/3R |  |  |  |  |  |
| CC+CT | 3R3R | 70 (56.0) | 56 (54.4) | 1.000 (reference) |  |  |
| CC+CT | 2R3R+2R2R | 40 (32.0) | 25 (24.3) | 0.813 (0.438―1.507) | 0.510 | 0.510 |
| TT | 3R3R | 12 (9.6) | 14 (13.6) | 1.402 (0.597―3.296) | 0.438 | 0.510 |
| TT | 2R3R+2R2R | 3 (2.4) | 8 (7.8) | 3.289 (0.829―13.06) | 0.091 | 0.273 |
| *MTHFR* 677C>T | *TS* 1494 0bp/6bp |  |  |  |  |  |
| CC+CT | 0bp0bp | 62 (49.6) | 46 (44.7) | 1.000 (reference) |  |  |
| CC+CT | 0bp6bp+6bp6bp | 48 (38.4) | 35 (34.0) | 1.053 (0.585―1.897) | 0.863 | 0.863 |
| TT | 0bp0bp | 8 (6.4) | 5 (4.9) | 0.874 (0.265―2.880) | 0.825 | 0.863 |
| TT | 0bp6bp+6bp6bp | 7 (5.6) | 17 (16.5) | 3.256 (1.245―8.512) | 0.016 | 0.048 |
| *MTHFR* 1298A>C | *TSER* 2R/3R |  |  |  |  |  |
| AA | 3R3R | 53 (42.4) | 43 (41.7) | 1.000 (reference) |  |  |
| AA | 2R3R+2R2R | 26 (20.8) | 23 (22.3) | 1.100 (0.550―2.202) | 0.787 | 0.787 |
| AC+CC | 3R3R | 29 (23.2) | 27 (26.2) | 1.202 (0.616―2.347) | 0.590 | 0.787 |
| AC+CC | 2R3R+2R2R | 17 (13.6) | 10 (9.7) | 0.725 (0.298―1.759) | 0.476 | 0.787 |
| *MTHFR* 1298A>C | *TS* 1494 0bp/6bp |  |  |  |  |  |
| AA | 0bp0bp | 47 (37.6) | 28 (27.2) | 1.000 (reference) |  |  |
| AA | 0bp6bp+6bp6bp | 32 (25.6) | 38 (36.9) | 2.052 (1.047―4.021) | 0.036 | 0.108 |
| AC+CC | 0bp0bp | 23 (18.4) | 23 (22.3) | 1.650 (0.777―3.506) | 0.193 | 0.290 |
| AC+CC | 0bp6bp+6bp6bp | 23 (18.4) | 14 (13.6) | 1.006 (0.441―2.294) | 0.988 | 0.988 |
| *TSER* 2R/3R | *TS* 1494 0bp/6bp |  |  |  |  |  |
| 3R3R | 0bp0bp | 53 (42.4) | 40 (38.8) | 1.000 (reference) |  |  |
| 3R3R | 0bp6bp+6bp6bp | 29 (23.2) | 30 (29.1) | 1.429 (0.737―2.771) | 0.290 | 0.701 |
| 2R3R+2R2R | 0bp0bp | 17 (13.6) | 11 (10.7) | 0.840 (0.353―1.997) | 0.692 | 0.701 |
| 2R3R+2R2R | 0bp6bp+6bp6bp | 26 (20.8) | 22 (21.4) | 1.148 (0.567―2.328) | 0.701 | 0.701 |
| Adjusted by age of female participants; RIF, recurrent implantation failure; RPL, recurrent pregnancy loss. | | | | | | |
