# Supplementary material for: Genetic Variation of Methylenetetrahydrofolate Reductase (MTHFR) and Thymidylate Synthase (TS) Genes Is Associated with Idiopathic Recurrent Implantation Failure
Source: PLoS One. 2016 Aug 25;11(8):e0160884. doi: 10.1371/journal.pone.0160884 (PMC4999086; doi:10.1371/journal.pone.0160884)
Supplement: S5 Table — (DOCX) [file pone.0160884.s005.docx]

| S5 Table. Allelic gene-gene interaction (2 sites) of one-carbon metabolism-related gene polymorphisms between controls and RIF patients. | | | | | |
| --- | --- | --- | --- | --- | --- |
| Haplotype | Controls (2n = 250) | RIF patients (2n = 240) | OR (95% CI) | *P* | FDR-*P* |
| *MTHFR* 677/*TSER* 238 | | | | |  |
| C-3R | 120 (48.0) | 114 (47.6) | 1.000 (reference) |  |  |
| C-2R | 36 (14.4) | 16 (6.5) | 0.468 (0.246 - 0.889) | 0.021 | 0.041 |
| T-3R | 81 (32.4) | 82 (34.0) | 1.066 (0.714 - 1.590) | 0.761 | 0.761 |
| T-2R | 13 (5.2) | 28 (11.8) | 2.267 (1.119 - 4.594) | 0.027 | 0.041 |
| *MTHFR* 677/*TS* 1494 | | | | |  |
| C-0bp | 120 (48.0) | 103 (43.1) | 1.000 (reference) |  |  |
| C-6bp | 36 (14.4) | 27 (11.1) | 0.874 (0.497 - 1.536) | 0.670 | 0.670 |
| T-0bp | 65 (26.0) | 66 (27.3) | 1.183 (0.768 - 1.822) | 0.509 | 0.670 |
| T-6bp | 29 (11.6) | 44 (18.5) | 1.768 (1.032 - 3.027) | 0.043 | 0.129 |
| RIF, recurrent implantation failure; *p*-value Fisher’s exact test. | | | | | |
