# Supplementary material for: Genetic Variation of Methylenetetrahydrofolate Reductase (MTHFR) and Thymidylate Synthase (TS) Genes Is Associated with Idiopathic Recurrent Implantation Failure
Source: PLoS One. 2016 Aug 25;11(8):e0160884. doi: 10.1371/journal.pone.0160884 (PMC4999086; doi:10.1371/journal.pone.0160884)
Supplement: S6 Table — (DOCX) [file pone.0160884.s006.docx]

| S5 Table. Meta–analysis of *MTHFR* 677C>T polymorphisms. | | |  |
| --- | --- | --- | --- |
| Study | Controls (TT/CC+CT) | RIF patients (TT/CC+CT) | OR (95% CI) |
| Azem F et al. (2004) | 4/40 | 8/37 | 2.162 (0.601―7.783) |
| Qublan HS et al. (2006) | 2/98 | 13/77 | 8.273 (1.812―37.76) |
| Safdarian L et al. (2014) | 1/94 | 11/85 | 12.165 (1.538―96.22) |
| Present study | 15/110 | 25/95 | 1.930 (0.962―3.873) |
| Total (fixed effects) | 22/342 | 57/294 | 3.084 (1.826―5.210) |
| Total (random effects) | 22/342 | 57/294 | 3.394 (1.451―7.938) |
